# The Role of Follow-up in Monitoring the Outcomes of Prematurity in a Cohort of Romanian Infants

**DOI:** 10.4274/balkanmedj.2015.1125

**Published:** 2017-01-05

**Authors:** Anca Bivoleanu, Andreea Avasiloaiei, Mihaela Moscalu, Maria Stamatin

**Affiliations:** 1 Regional Neonatal Intensive Care Unit, Grigore T. Popa University of Medicine and Pharmacy, Cuza-Voda Clinical Hospital of Obstetrics and Gynaecology, Iasi, Romania; 2 Regional Neonatal Intensive Care Unit, Division of Neonatology, Department of Mother and Child Care, Grigore T. Popa University of Medicine and Pharmacy, Cuza-Voda Clinical Hospital of Obstetrics and Gynaecology, Iasi, Romania; 3 Department of Preventive Medicine and Interdisciplinary Sciences, Grigore T. Popa University of Medicine and Pharmacy, Iasi, Romania

**Keywords:** Neurologic sequelae, prematurity, infants, monitoring

## Abstract

**Background:**

The rate of preterm births in Romania is one of the highest among European countries. However, there is little information regarding the ways in which premature birth affects the outcome in Romanian preterm infants.

**Aims:**

To investigate the effects of early developmental intervention after discharge from the hospital on motor and cognitive development in preterm infants.

**Study Design:**

Longitudinal observational study.

**Methods:**

We performed the Amiel-Tison neurologic evaluation at discharge and the Bayley Scales of Infant Development from 3 to 24 months. Based on these evaluations, an outcome score was formulated.

**Results:**

Between 2007 and 2010, 1157 of 2793 premature infants were included into the study. There was a negative correlation between the number of evaluations and the risk of developing neurologic sequelae (p<0.001). The correlation analysis demonstrated a significant association between the final category of risk at the end of the follow up program and the degree of compliance (p<0.01). At 24 months evaluation, there was a correlation between the low gestational age and the risk of developing severe neurologic sequelae (p<0.001).

**Conclusion:**

This study shows the importance of follow up program in decreasing the risk of developing neurologic sequelae in preterm infants.

Prematurity involves complex medical conditions (mainly respiratory, but also infectious, gastrointestinal, feeding related and growth related), in addition to neurodevelopmental morbidities (neurologic, motor, sensory and behavioral), the severity of which increases with decreasing gestational age. These conditions affect the risk of morbidity and mortality, and they significantly influence later quality of life (1,2,3,4). 1 in 10 premature infants will develop a permanent minor neurological dysfunction, such as language disorders, learning disabilities, attention deficit-hyperactivity disorder, neuro-motor dysfunction or developmental coordination disorders, behavioral problems, and social-emotional difficulties, but also chronic health issues such as asthma, infections and increased risk of Sudden Infant Death Syndrome (5,6,7,8,9).

Due to injury to the developing brain, 50% of premature infants born before the 26th week of gestation may present major neurological sequelae (e.g., cerebral palsy (CP), mental retardation, visual and hearing deficits) and minor issues (e.g, impaired cognitive abilities and physical skills, learning difficulties, depression, anxiety, or difficulty interacting with other children their age) (10,11,12,13). Therefore, it is essential for follow-up services to provide medical, developmental evaluation and support for this category of patients.

In Romania, the rate of preterm births is 9%, almost twice as high as in the majority of European countries (14,15). Because in our country little information is available regarding the ways in which premature birth affects outcome, we sought to estimate the role of follow-up programs and early developmental intervention in evaluating the degree of risk for neurologic sequelae in a cohort of premature infants from our Maternity Hospital.

## MATERIALS AND METHODS

### Study population

This longitudinal observational study was conducted on 1157 preterm infants from January 1, 2007 to December 31, 2010 at a tertiary-level Neonatal Intensive Care Unit in Iasi, Romania. Inclusion criteria were in accordance with our national guidelines for follow-up of high-risk neonates (16): preterm birth, birth weight of less than 1500 grams, perinatal asphyxia with Apgar scores of 3 or less at 5 minutes and/or signs of hypoxic-ischemic encephalopathy, mechanical ventilation for more than 24 hours, infants with central nervous system injury (intraventricular hemorrhage, periventricular leukomalacia, seizures, hydrocephalus, encephalopathy), extracranial or intracranial trauma, respiratory disorders, including bronchopulmonary dysplasia, necrotizing enterocolitis, infections-meningitis and/or culture positive sepsis, hyperbilirubinemia that requires exchange transfusion, fetal growth restriction, multiple preterm births, metabolic issues-symptomatic hypoglycemia and hypocalcemia, abnormal neurological examination at discharge, social/environmental conditions (low economic status, underage mothers, drugs/alcohol abuse, smoking).

Infants with major congenital anomalies and infants requiring major surgery were excluded. Perinatal data were collected prospectively during admission, stored in the neonatal intensive care unit database, and retrospectively retrieved for data analysis. Parental informed consent was obtained for participation in the follow-up program and inclusion in the present study. The hospital’s Committee of Ethics approved of our research.

### Survey protocol

At discharge, or as close as possible to the postmenstrual age of 40 weeks, the neonatal neurological examination was performed according to the Amiel-Tison protocol, by the same trained neonatologist, who examined all infants included in this study. The Amiel-Tison test (17,18) involves 35 items covering neurosensory aspects, cranial morphology, passive and active muscle tones, spontaneous motor activity and primary reflexes. The scoring system is based on a three-point ordinal scale where ‘0’ corresponds to a typical response, ‘1’ to a moderately abnormal response, and ‘2’ to a definitely abnormal response. Items that have neuropsychological relevance include: neurosensory aspects (alertness, visual fixation), cranial morphology (head circumference, anterior fontanels, squamous sutures and other sutures), passive tone in limbs and axis, rapid stretching, active tone and primitive reflexes. The infants were placed in three risk categories [low (L), moderate (M), severe (S)], based on the number of abnormal examination findings.

At 3, 6, 9, 12, 18 and 24 months corrected age, we performed the Bayley Scales of Infant Development, 2^nd^ Edition (BSID II) (19-21). All developmental milestones are assessed according to corrected age, in order to compensate for prematurity. The results were summarized into ﬁve domains: cognitive development, neuro-motor development, language development, behavior and general health. During regular evaluations, according to total number of items failed, we placed the infants in three risk categories (L, M, S). If the infant had on-going issues or illnesses, such as delays in reaching developmental milestones, we recommended more frequent visits and physiotherapy. Mothers were also instructed to perform their infants’ specific daily exercises. As needed, the patient was referred to another specialist for early intervention services and/or to recommend further evaluation and therapy.

According to the total number of items failed during regular evaluations and at the end of the follow-up program, we included the infants in specific risk categories: L, M or S.

We considered the patient to be low risk if the patient presented with minor hypotonia or hypertonia without other neurologic abnormalities (cognitive and learning deficits, behavioral and emotional abnormalities) and/or minor language delay. M risk was defined as mild deviation in muscle tone regulation, reﬂexes, ﬁne or gross motor performance, moderate cognitive and/or speech or language disorders. Major risk was defined as severe neurodevelopment dysfunctions: CP, mental retardation, visual impairment and/or hearing loss. For this study, we chose gestational age, birth weight, respiratory distress syndrome, intracranial hemorrhage, and periventricular leukomalacia in order to examine the correlation between these entities and the category of risk.

Because the infants with regular evaluations were referred to special rehabilitation programs, in order to correlate compliance with the follow-up program and risk categories (L/M/S-according to the BSID II examination), we created an “outcome score”, as follows:

“0” if the infant at the final evaluation, at two years corrected age, maintained the same risk category, (L/M/S)

“1”, if the infant at the final evaluation, progressed from moderate to L risk or from S to M,

“2” for those infants, who passed at the final evaluation directly from S risk to L risk,

“-1” if the infant at the final evaluation, regressed from L risk to M and/or from M to S risk,

“-2” for patients who, at the final evaluation, passed directly from L to S risk.

### Statistical analysis

The data were analyzed using SPSS version 20.0 (SPSS Inc.; Chicago, IL, USA). Data were expressed as mean, standard deviation (SD), median, and interquartile range, or as per cent frequency, as appropriate. Comparisons among groups were made by p value for linear trends (1-way analysis of variance or chi-square test). Among patients, comparisons were made by paired t test (for normally distributed data) or by Wilcoxon signed rank test (for non-normally distributed data). Correlations between variables were investigated using the Pearson product moment correlation coefficient (r), contingency coefficient, or by Spearman Rank correlation coefficient, as appropriate. Statistical significance was defined as p<0.05.

## RESULTS

**Figure 1:**
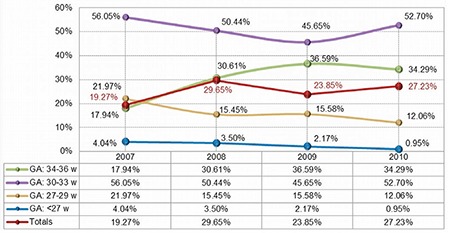
The incidence of prematurity among study subjects.GA: gestational age

Over the four-year period of this study, 4116 infants were admitted to our hospital. 67.9% (n=2793) were premature and 41.4% (n=1157) were enrolled in the follow-up program and in this study. 2.4% (n=28) of patients died after discharge and before completing the follow-up program-2 years corrected age. The percentage of premature infants among those admitted to the hospital increased during the study period, from 19.3% in 2007, to 27.2% in 2010 (Figure 1).

Mean gestational age was 31.9 (±2.0 SD) weeks. The frequency of infants with gestational age less than 34 weeks decreased significantly, while the frequency of infants with gestational age over 34 weeks increased during the studied period (F=7.45, p=0.00006, 95% CI). The mean birth weight was 1720 (±541 SD) grams.

When evaluated by the Amiel-Tison neurologic examination tool at discharge, 47.3% of infants were found to be L risk; 41.7% were M risk; 11.1% were S risk.

Mothers/families were informed about the significance and importance of the follow-up program and were asked to return for periodic evaluations according to a personalized schedule. 72.7% (n=841) came for all 6 recommended evaluations, 20.3% (n=235) came 2-5 times for evaluation and 7.0% (n=81) came for only one examination, and therefore their results were analyzed based only according to this single evaluation. Of those with six evaluations, the highest percentage was represented by infants of gestational age 34-36 weeks: 42.0%. The results indicate a significant association between gestational age of infants and the frequency of evaluations during the follow-up program (χ^2^=36.014, r=0.4627, p=0.00247, 95% CI).

At every stage of the follow-up evaluation, we changed the respective category of risk according to the infant’s performance. The highest percentage of infants with severe (8.7%) and medium risk (62.8%) was at three month corrected age (Table 1). The rate of death correlated significantly with low gestational age, as proven by the non-parametric analysis (χ^2^=18.64, r=0.508, p=0.00317, 95% CI).

According to the BSID II evaluation, at the end of the follow-up program, 3.6% were included in the high-risk group, and 26.3% were placed in the medium-risk group; 68.5% were considered to have a L risk of developing subsequent neurologic disabilities.

**Figure 2:**
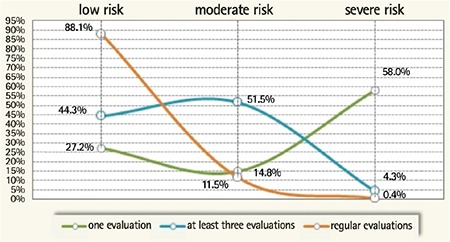
Correlation between the degree of risk and number of evaluations.

Premature infants that presented for all six recommended evaluations made up the highest percentage of infants at L risk (88.1%), as compared to those with a single evaluation (27.2%). The present study highlights a significant correlation between the number of evaluations and the risk in developing neurological sequelae (χ^2^=35.19, r=0.709, p=0.000142, 95% CI) (Figure 2).

**Figure 3:**
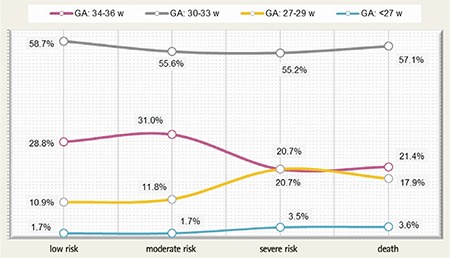
Distribution of preterm infants according to the degree of risk and gestational age. GA: gestational age

The association between gestational age and the degree of estimated risk observed at the end of the follow-up program demonstrates a significant correlation between low gestational age and a S risk of developing neurological sequelae (χ^2^=27.008, r=0.629, p=0.00014, 95% CI) (Figure 3). All patients with S risk at discharge and only one evaluation were deceased.

Regarding the infants who attended all six evaluations, for the high-risk category, according to the Amiel-Tison examination, 68.0% passed from S risk to M; 3.0% passed from S risk t L risk; 2.9% remained in the S risk category.

**Figure 4:**
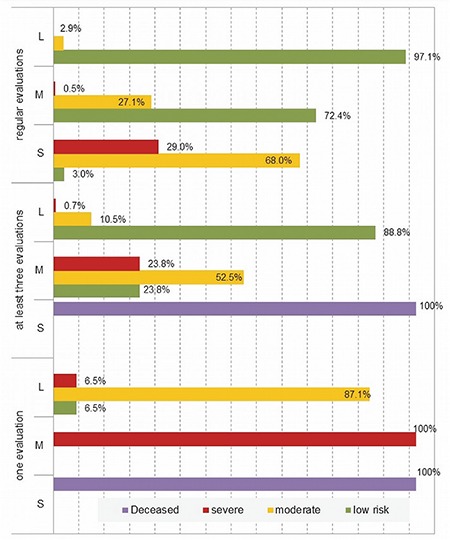
Amiel-Tison examination and BSID II examination versus number of evaluations and degree of risk.L: low; M: moderate; S: severe

Among those with M risk, 27.1% remained in this category, 72.4% reached L risk and only 0.5% moved into the S risk category. Furthermore, 97.1% of patients found with L risk at discharge remained in this category, and only 2.9% passed into M risk (Figure 4). There was a significant correlation between the result of the final examination at 2 years corrected age and the Amiel-Tison examination and the frequency of evaluations (χ^2^=339.3, p<0.01).

According to our “outcome score”, 4.94% of those with one evaluation and 0.43% with at least three received scores of “-2”. “-1” received 70.37%, those with one evaluation, 11.49% with least three evaluation and only 1.07% with regular evaluations, “0” score - 24.69% from those with one evaluation, 80% with minimum 3 evaluations and 56.12% with regular evaluations, “+1”, no one from those with one evaluation, 8.09% with least three evaluations and 42.45% with regular evaluations and “+2”, no one from those with one and least three evaluation and 0.36% from those with regular evaluations (Table 2).

Correlation analysis demonstrated a significant association between the final category of risk at the end of the follow-up program and the degree of compliance (χ^2^=618.70, p<0.001, 95% CI).

## DISCUSSION

Since the early 1970s, it has been known that the neonatal neurological examination is valuable in predicting the future development of an infant. An abnormal result of the examination has been shown to correlate with an abnormal developmental outcome in preterm infants (1,4,5). Monitoring the attainment of developmental milestones is essential for the early diagnosis of developmental disabilities, in order to institute early intervention and influence the quality of life for these patients.

According to the National Guidelines for Follow-up of the High-Risk Neonate, in Romania, this program is performed only in Level III maternity hospitals, up to the age of two years (chronologic for term infants and corrected for premature infants). In our neonatal intensive care unit, the follow-up program was implemented in 2006. The Amiel-Tison neurological examination test was performed at discharge, as close as possible to 40 weeks of gestation and the BSID II was performed during the two years of the follow-up program. Despite all the limitations and controversies surrounding this assessment tests, they do form an effective means of identifying infants with delayed development, so that early intervention can be started.

The region of Moldova has a low socio-economic status. Our maternity services extend throughout this entire area. The incidence of premature babies admitted in our unit increases from 19.27% to 27.23% over the studied period. This high percentage can be explained also by the fact that our hospital is a referral center for both high-risk pregnancies and sick preterm infants. The regional enrollment of a study population, despite the low socio-economic status of our region, points out a reasonable willingness of families to participate in the follow-up program (in our study, 72.7% came for regular evaluations). A reliable follow-up program depends on maintaining a high rate of participation.

Our study revealed a higher incidence of neurodevelopmental disabilities (29.9%), compared with other data (18), probably due to the low social and economic status of the infants’ families.

Social and/or environmental characteristics of families and the mother-child interaction are predictive factors for later outcome (22,23). Low socio-economic status was found to be an independent risk factor in the study conducted by Ruth et al. (24). The main limitation of our cohort study is the absence of these correlations. Although we acknowledge the major influence of factors such as initial condition, severity of neurologic injury or gestational age, we focused on the correlation between compliance to the follow-up program and neurologic risk outcome.

Gestational age is a decisive factor in motor, cognitive and behavioral development, in addition to intensive recovery therapy; the percentage of premature infants at S risk is inversely proportional to gestational age. Normal neurological assessment of preterm infants at 40 weeks corrected age, together with a gestational age of more than 34 weeks of gestation and no severe cerebral lesions, is associated with a signiﬁcantly lower risk of suboptimal neurologic development at 2 years of corrected age (24).

In this study, the highest percentage (42%) with L risk and regular evaluation was represented by infants with gestational age of 34-36 weeks. We might explain this by the fact that mothers were encouraged by the progressive favorable evolution of their infants. Meanwhile, only 2.4% of infants less than 27 weeks of gestation came for all 6 recommended evaluations.

At the moment of hospital discharge, the neurological examination of high-risk premature infant is not decisive in predicting neurodevelopmental disabilities; however, certain abnormal findings are associated with abnormal neurologic acquisitions. By the age of 2 years, major handicaps such as gross motor disturbances and severe mental retardation will have become clear, but subtle neurological impairment, behavioral and learning difficulties, especially in former high-risk premature infants are often noticed at school age.

Due to the high rate of prematurity in Romania and the low socio-economic status of the majority of the population, an extended program of follow-up, at least up to 7 years of age, is badly needed, especially since some neurocognitive, executive and behavioral malfunctions can only be detected at school age and preterm birth has an effect into adulthood (25,26,27,28,29,30).

## CONCLUSION

Gestational age remains the leading factor in including premature infants in a specific group of developmental risk. Our results demonstrate, importantly, that the category of risk can improve over time with repeated evaluations and specific therapy. S risk was 11.1% at discharge and 5.1% at 24 months corrected age (the end of the follow-up program). Because major developmental disabilities are likely to be found before 2 years of age, it can be assumed that the incidence of infants with high risk is not underestimated.

The present study highlights the strong correlation between regular evaluations and a decreased risk of developing neurological sequelae. This emphasizes the importance of follow-up programs for the early identification of the degree of risk, as well as the importance of involving the patients’ families in order to improve the neurologic outcomes of their children.

## Figures and Tables

**Table 1:**
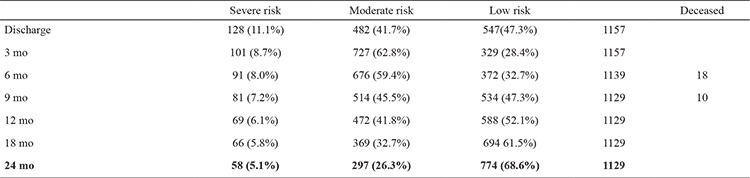
Evaluations by age and risk categories

**Table 2:**
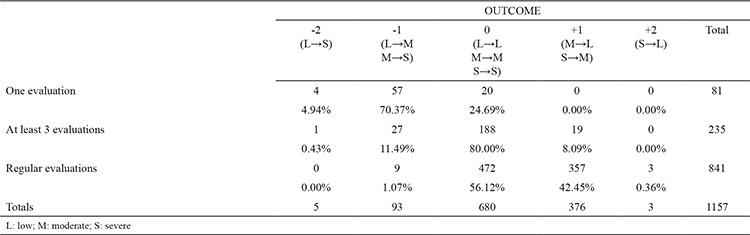
Outcome scores
